# Carcinoid Heart Disease: A Classic One, But Not Always Remembered

**DOI:** 10.7759/cureus.77932

**Published:** 2025-01-24

**Authors:** Pamela Ramírez-Rangel, Jorge D Rodríguez-Esparza, Xochitl A Ortiz-Leon, Joel E Vargas-Ahumada, Roberto Herrera-Goepfert

**Affiliations:** 1 Adult Cardiology Department, Instituto Nacional de Cardiología Ignacio Chávez, Mexico City, MEX; 2 Echocardiography Laboratory, Instituto Nacional de Cardiología Ignacio Chávez, Mexico City, MEX; 3 Nuclear Medicine and Molecular Imaging Department, Instituto Nacional de Cancerología, Mexico City, MEX; 4 Surgical Pathology Department, Instituto Nacional de Cancerología, Mexico City, MEX

**Keywords:** carcinoid heart disease, multimodality cardiac imaging, neuroendocrine tumor, pulmonary regurgitation, tricuspid regurgitation

## Abstract

A 24-year-old female presented a dermatosis characterized by confluent erythematous spots on her face and neck and was diagnosed with presumed systemic lupus erythematosus. Five years later, she was admitted to our Cardiology Department due to symptoms of right heart failure (fatigue, dyspnea, edema in inferior limbs, and ascites). Thickened, stiff tricuspid and pulmonary valve leaflets that caused severe tricuspid and pulmonary regurgitation were seen on transthoracic echocardiography. Cardiac magnetic resonance showed right heart dilatation and right ventricular dysfunction. Multimodal imaging with contrast-enhanced computed tomography and dual positron emission tomography (PET/CT) with (^18^F) fluorodeoxyglucose and (^18^F) AlF-NOTA-octreotide showed liver lesions highly suggestive of metastasis. The liver biopsy confirmed a well-differentiated neuroendocrine tumor. A diagnosis of carcinoid heart disease (CHD) was made. She was treated with lanreotide (a long-acting somatostatin analog) and surgical replacement of the tricuspid and pulmonary valves.

## Introduction

Neuroendocrine tumors (NETs) are rare neoplasms with an incidence of 2.5 to five cases per 100,000 [1]; those that originate in the gastrointestinal tract are referred to as carcinoids. NETs have an indolent evolution; after a prolonged natural history of the disease, the patient debuts with carcinoid syndrome (CS), which is a common complication, with an incidence of about 30-40% in patients with metastatic disease [2]. In most cases, it is caused by the secretion of bioactive amines (mainly serotonin) by the enterochromaffin cells of a large tumor or liver metastases. Symptoms of CS typically do not emerge until liver metastases have severely affected liver function, as serotonin undergoes degradation in the liver by hepatic monoamine oxidase. Symptoms include skin flushing, diarrhea, bronchospasm, and fibrotic valvular heart disease [3]. The development of valvular heart disease is due to the excessive release of vasoactive substances from NETs, including serotonin, prostaglandins, histamine, tachykinin, and kallikrein. The tumor-released 5-hydroxytryptamine (5-HT) interacts with the 5-HT2B subtype receptors in the heart valves. This interaction leads to an inflammatory reaction and an increase in transforming growth factor beta 1. This results in the accumulation of plaque-like material on the endocardial surfaces of the valve leaflets and the subvalvular apparatus. Such plaque deposits primarily cause fibrotic valvular heart disease in the right side of the heart since vasoactive substances such as serotonin are metabolized in the pulmonary vasculature, preventing their transport to the left heart [2]. The following case shows the evolution of carcinoid heart disease (CHD) that involves the tricuspid and pulmonary valves, leading to congestive heart failure in a young patient who underwent surgical management.

## Case presentation

**Figure 1:**
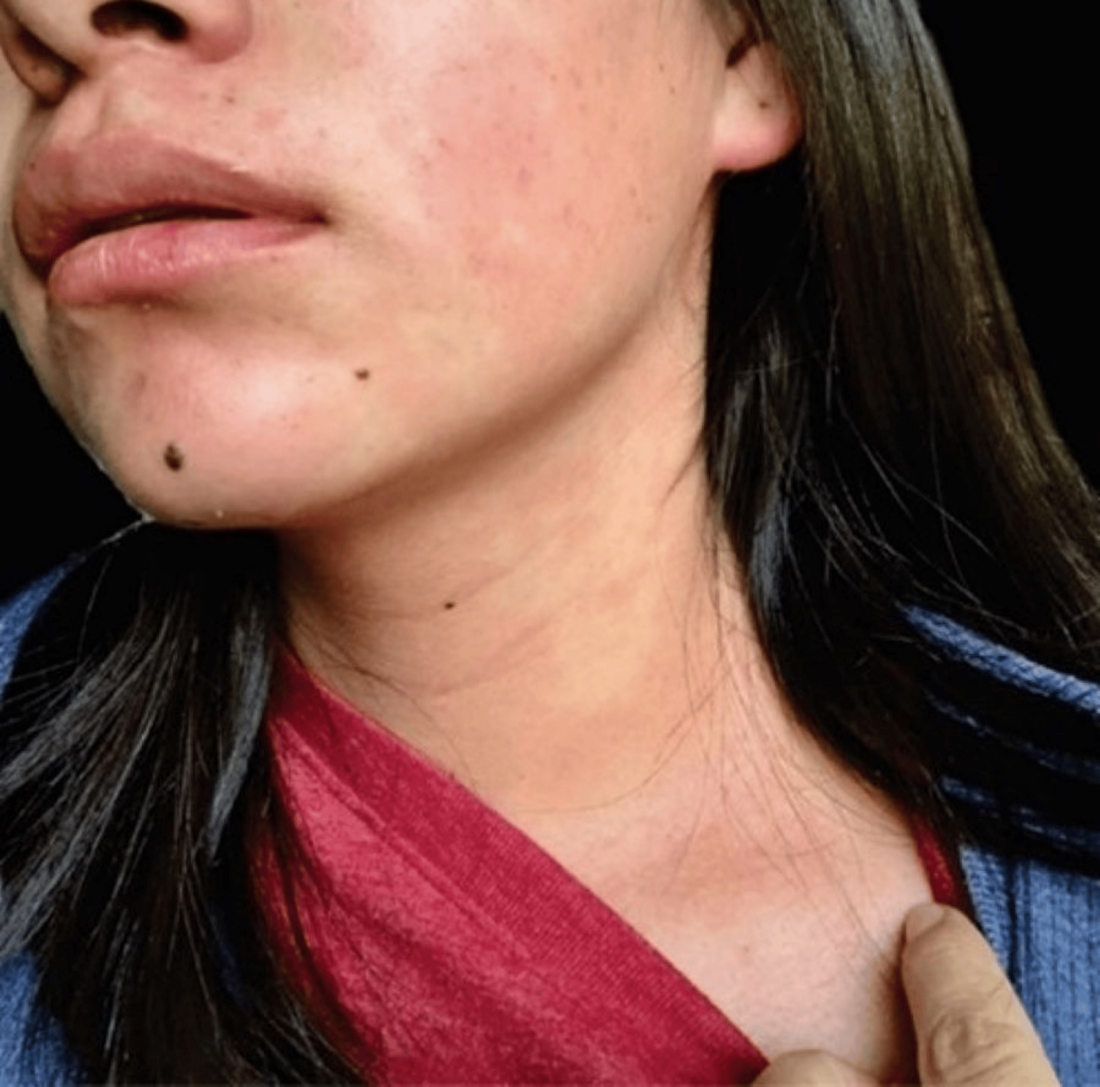
Dermatosis Dermatosis that affects the face and neck consists of convergent erythematous spots with irregular and defined edges. Facial rash is one of the most characteristic manifestations of carcinoid syndrome.

A 24-year-old female presented facial flushing (Figure 1) and diarrhea five years early and was diagnosed with presumed systemic lupus erythematosus in a general hospital. Then, fatigue, dyspnea, edema in inferior limbs, and ascites appeared. The diagnosis of heart failure was considered, and she was admitted to our Cardiology Department. The vital signs were as follows: blood pressure was 100/60 mmHg, pulse rate was 78 beats/min, breathing rate was 25 breaths/min, and oxygen saturation was 92% to ambient air. On physical examination, an elevated jugular venous pulse with prominent V waves was observed, indicative of right ventricular volume overload, right atrial pressure elevation, and superior vena cava dilatation resulting in jugular venous congestion. A pan-systolic murmur was heard at the left middle sternal border, accentuated by deep breathing typical of tricuspid regurgitation, and auscultated. An early diastolic murmur of pulmonary regurgitation was also present.

**Figure 2:**
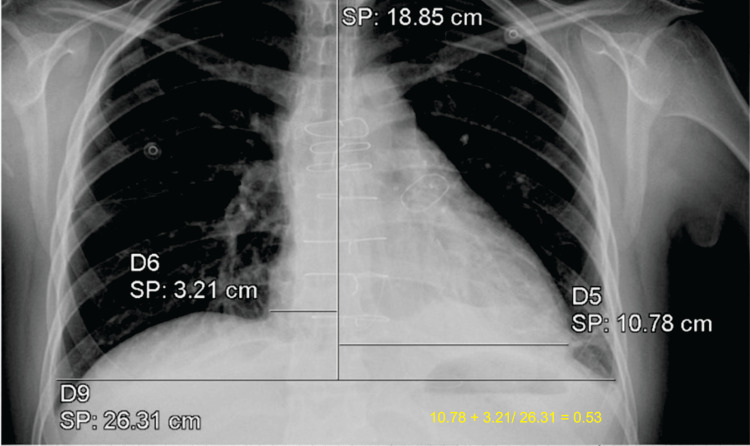
Chest radiography Chest radiography showed cardiomegaly stage 1 with a cardiothoracic index of 0.53.

**Figure 3:**
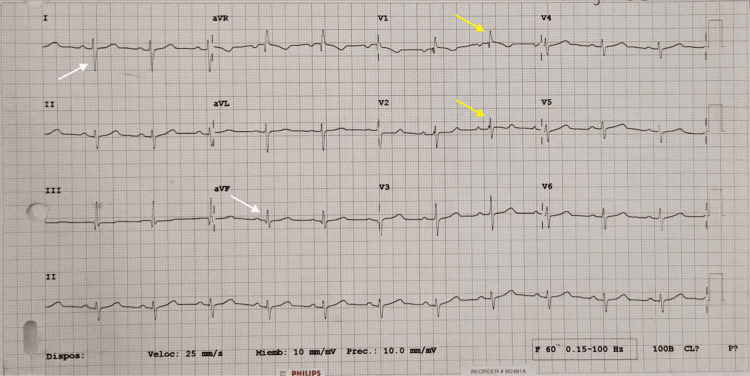
Electrocardiogram Electrocardiogram shows sinus rhythm, right-axis deviation (150°) (white arrows), rsR’ and rR’s patterns in V1 and V2, respectively (yellow arrows); QRS length of 120 milliseconds; and a Cabrera index of 0.7.

Chest radiography showed cardiomegaly (Figure 2), while in the electrocardiogram, an incomplete right bundle branch block and right ventricle hypertrophy were observed (Figure 3). The laboratory results revealed an N-terminal pro-B-type natriuretic peptide (NT-pro-BNP) level significantly increased (3,454 pg/mL, reference range: less than 125 pg/mL); the rest of the tests were normal.

**Figure 4:**
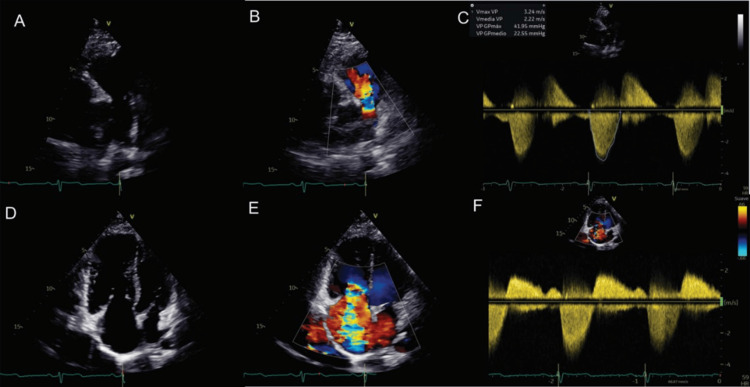
Transthoracic echocardiography A) A parasternal short axis view focused on the pulmonary valve shows the thickening of the cusps and limitation of their mobility. B) Color Doppler echocardiography displays a wide jet of pulmonary regurgitation (PR). C) Evaluation of the pulmonary valve by continuous wave Doppler compatible with moderate stenosis (peak velocity 3.2 m/s, peak gradient 41 mmHg) and severe regurgitation (PR index of 0.65). D) An apical four-chamber right ventricle focused view demonstrates thickening of the tricuspid leaflets mainly on the tips and a wide coaptation gap in systole. D) Color Doppler echocardiography shows a severe tricuspid regurgitation (TR) jet. E) Evaluation by continuous wave Doppler. A dense triangulated regurgitant jet consistent with severe TR is observed; the mean pressure gradient (4 mmHg) and the pressure half-time (173 milliseconds) were consistent with moderate tricuspid stenosis.

**Figure 5:**
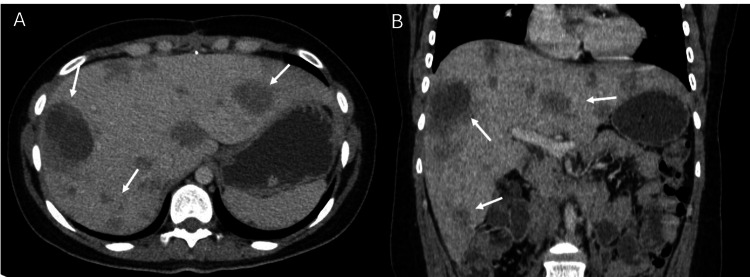
Contrast-enhanced computed tomography Contrast-enhanced computed tomography in the axial (A) and coronal (B) reconstruction in the portal venous phase. Multiple nodular hypodense images are visualized in all liver segments (white arrows), highly suggestive of metastasis.

**Figure 6:**
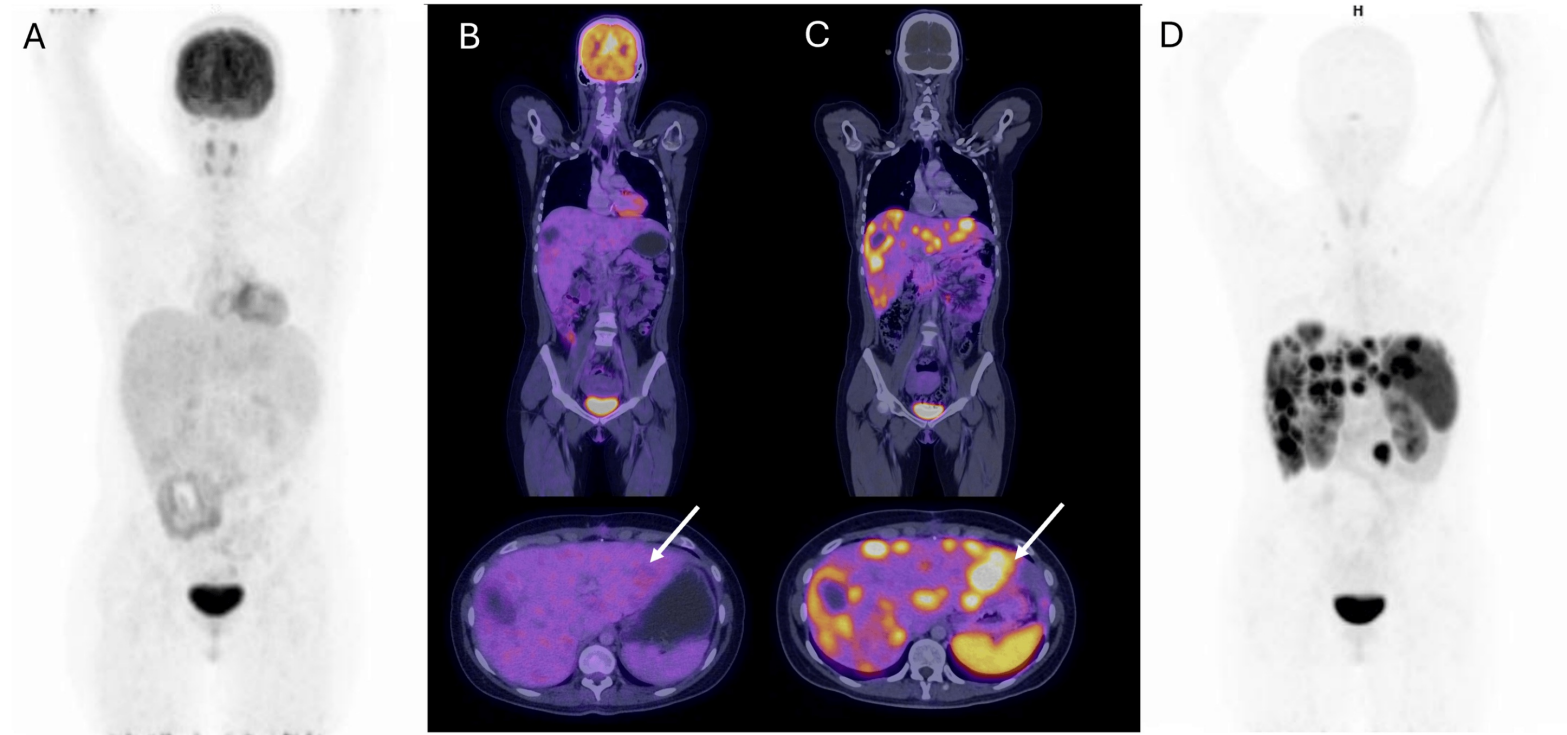
Dual positron emission tomography fused with computed tomography Dual positron emission tomography fused with computed tomography (PET/CT) A) PET/CT with (^18^F) Fluorodeoxyglucose (FDG) maximum intensity projection (MIP) with no sites of abnormal uptake of the radiotracer (B). ^18^F-FDG PET/CT axial and coronal fused images show multiple liver lesions without metabolic activity (white arrow). (C) (^18^F) AlF-NOTA-octreotide PET/CT axial and coronal fused images; multiple liver lesions are identified with overexpression of somatostatin receptors (white arrow). (D) (^18^F) AlF-NOTA-octreotide PET/CT MIP shows multiple positive hepatic lesions; a focal uptake of the radiotracer was also observed in small intestine (proximal ileum). The dual image findings correspond to well-differentiated neuroendocrine neoplasia.

**Figure 7:**
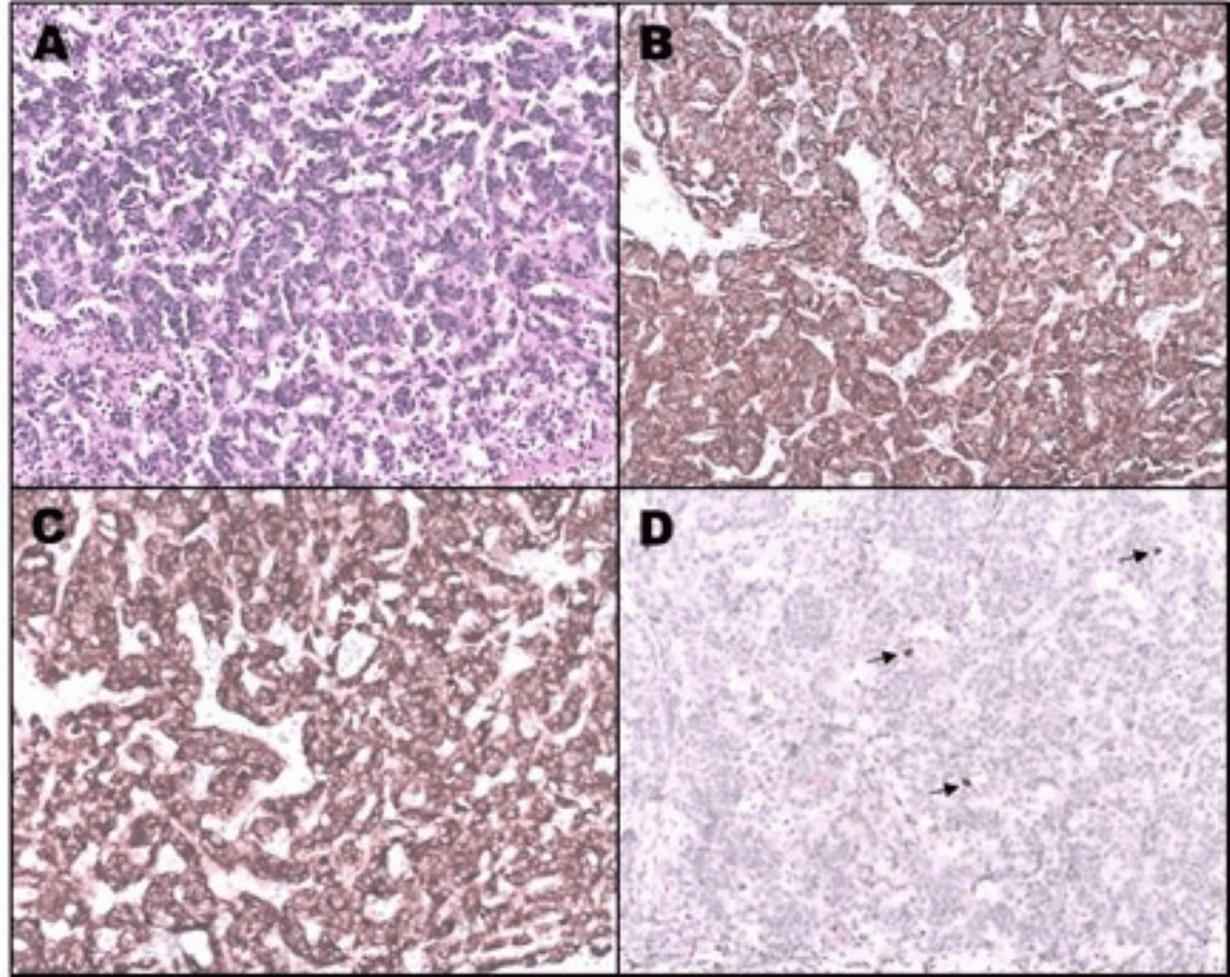
Pathology Cords and retracted groups of small cells with hyperchromatic nuclei and scant cytoplasm are observed, surrounded by dense vascularized fibroconnectic tissue (A; hematoxylin and eosin, 10x). The immunoreactions for chromogranin (B; 10x) and synaptophysin (C; 10x) are positive in the cytoplasm of the neoplastic cells. The nuclear proliferation index (Ki-67; arrows) was less than 2% (D; 10x).

The transthoracic echocardiogram (TTE) showed right atrial and ventricular dilation, thickened, retraction, and reduced mobility of the tricuspid and pulmonary valve leaflets, which caused severe regurgitation and moderate stenosis of both valves (Figure 4 and Video 1). Conventional echocardiographic parameters of right ventricular function were in normal ranges. Cardiac magnetic resonance imaging (CMRI) was done as a complementary evaluation and showed right heart dilatation due to volume overload, right ventricular dysfunction (ejection fraction of 42%), tricuspid and pulmonic rigid valves, and multiple liver lesions. In contrast-enhanced computed tomography (CT), multiple nodular hypodense lesions were visualized in all liver segments, highly suggestive of metastasis (Figure 5). Dual positron emission tomography (PET/CT) was performed with (^18^F) fluorodeoxyglucose and (^18^F) AlF- NOTA-octreotide showing molecular imaging findings of a well-differentiated neuroendocrine neoplasia (Figure 6). The liver biopsy confirmed a chromogranin +, synaptophysin +, Ki67 <2% well-differentiated neuroendocrine tumor; the small intestine was suggested as the primary site of the tumor (Figure 7).

**Video 1:** 3D valve reconstruction Three-dimensional reconstruction of the tricuspid valve presented as viewed from the right atrium. Thickening of the three leaflets and decreased valve opening orifice are observed. The septal and posterior leaflets are fixed.

In summary, the patient presented with symptoms of right heart failure (fatigue, dyspnea, edema in inferior limbs, and ascites). On physical examination, signs of jugular venous congestion and murmurs of tricuspid and pulmonary regurgitation were found. Chest radiography showed cardiomegaly, while the electrocardiogram demonstrated an incomplete right bundle branch block and right ventricular hypertrophy. The laboratory results revealed an NT-pro-BNP level significantly increased. TTE confirmed right heart chamber dilatation and severe tricuspid and pulmonary regurgitation due to thickened, retraction, and reduced mobility of both valves. An organic cause of valvular lesions was suspected. In the CMRI reduced right ventricular function was confirmed, and liver lesions were also found. The contrast-enhanced CT suggested that liver lesions were metastatic. The dual PET/CT showed molecular imaging findings of a well-differentiated neuroendocrine neoplasia that was confirmed by the liver biopsy. The diagnosis of CHD caused by a neuroendocrine tumor was made. The symptoms of flushing and diarrhea that the patient has been reporting for five years were attributed to CS.

**Figure 8:**
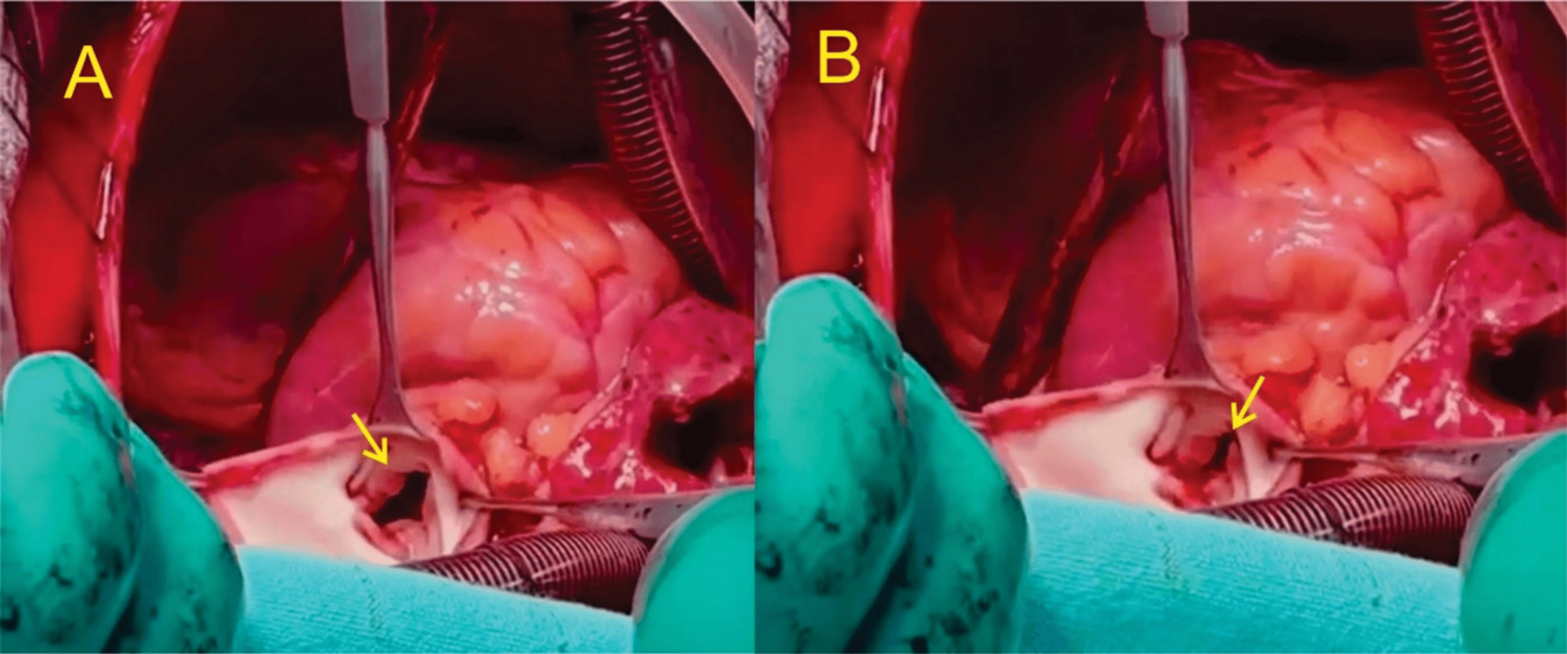
Trans-operative pulmonary valve Trans-operative view of the pulmonary valve (arrow). Thickened leaflets with maximum opening (A) and closing (B). Note that there is no coaptation of the leaflets, which was the cause of the severe pulmonary regurgitation observed in the echocardiogram and explained the early pulmonary regurgitation murmur auscultated on physical examination.

The patient was treated with lanreotide. The somatostatin analogs, octreotide (short-acting) and lanreotide (long-acting), are the most widely used medical therapy to treat CS symptoms and prevent a CS crisis. Both agents are synthetic octo-amino acid peptides that bind to the somatostatin receptor subtypes 2 and 5, inhibit serotonin release by the tumor, and reduce the secretion of gastrointestinal hormones, such as gastrin, secretin, and cholecystokinin. Diuretics were started to treat symptoms of right heart failure and serum serotonin levels were controlled to avoid a CS crisis, then the patient underwent tricuspid and pulmonary valve replacement with bioprosthetic valves (Figure 8). The selection of valve prostheses remains a matter of debate. The implantation of a mechanical prosthesis requires anticoagulation therapy, which raises the bleeding risk in patients with liver metastases. Moreover, mechanical prostheses in the tricuspid position increase the risk of thrombosis [4]. For these reasons, a bioprosthetic valve was preferred.

## Discussion

NETs are heterogeneous neoplasms that often arise in the gastrointestinal tract (carcinoids). Patients with well-differentiated NET of midgut organs in advanced stages may develop CS, which manifests with vasomotor changes, facial flushing, diarrhea, bronchospasm, and fibrotic heart disease [1].

CHD has a prevalence of 40-50% in patients with CS mainly seen in small bowel NETs. This condition is linked to significant morbidity and mortality with a three-year survival rate of only 31% without treatment [1].

The development of CHD is mainly due to the excessive release of vasoactive substances from NETs. The tumor-released 5-hydroxytryptamine interacts with the 5-HT2B subtype receptors located in the heart valves, initiating a mitogenic response in smooth cells and fibroblasts, leading to an inflammatory process and upregulation of transforming growth factor b1, resulting in the deposition of plaque-like materials (composed of myofibroblasts, smooth cells, and extracellular matrix) on the endocardial surface of the valve leaflets, subvalvular apparatus, cardiac chambers, the intima of pulmonary arteries, aorta, and vena cava; these plaque-like deposits mainly affect the right side of the heart in 90% of the cases with involvement of the tricuspid and pulmonary valves (as seen in this case) [2]. The fibrotic infiltrate retracts and decreases the mobility of the leaflets, particularly during the closure, originating valvular regurgitation, which leads to progressive right ventricular volume overload and, if not treated, right ventricular dysfunction. More than 95% of patients with valvular involvement have tricuspid valve disease, with roughly 90% showing moderate to severe tricuspid regurgitation. Among those with pulmonary valve disease, which affects around 85% of patients, approximately 80% experience regurgitation, while 50% have stenosis [4]. Vasoactive substances such as serotonin are metabolized in the pulmonary vasculature, preventing their transport to the left heart. Consequently, left-sided heart disease typically occurs only in conjunction with a right-to-left intracardiac shunt.

Predictors factors of the development of CHD in patients with CS are not completely understood but can include high liver tumor burden and high levels of serotonin [5].

Clinical manifestations of CHD include those of valvular heart disease and right heart failure: dyspnea, ascites, peripheral edema, pleural effusion, pulsatile hepatomegaly, and elevated jugular venous pulse with prominent V waves. A holosystolic murmur, best heard at the left middle sternal border, is commonly auscultated, which corresponds to tricuspid regurgitation. Concomitant murmurs of pulmonary stenosis or regurgitation may also be present. Clinical findings of pulmonary insufficiency typically include an early diastolic murmur at the left parasternal border. The reverse pressure gradient from the pulmonary artery to the right ventricle progressively decreases throughout the diastole and accounts for the decrescendo nature of the diastolic murmur.

The lack of cardiac signs and symptoms until advanced CHD indicates the need for screening for patients. Urinary 5-hydroxyindoleacetic acid (5-HIAA) and NT-pro-BNP should be performed; with a cut-off level of >300 µmol/24 hours and >260 ng/L, respectively, TTE is indicated [2]. In patients with CS, the sensitivity and specificity of NT-proBNP at a cutoff level of 260 pg/mL for the detection of CHD are 92% and 91%, respectively, whereas the sensitivity of urine 5-HIAA is 35-73%, and the specificity is 89-100% depending on the cutoff used [6]. A 24-hour urinary 5-HIAA level >300 mmol/24 hours is a useful marker for identifying those at risk of developing CHD. NT-proBNP seems to be the best biomarker to date for screening of clinically significant CHD in patients with CS. In addition, there is a significant correlation between NT-pro-BNP levels with the echocardiographic severity score and NYHA functional class [7]. NT-proBNP is secreted in response to stretching of the cardiac muscle due to increased pressure and thereby reflects the consequences of CHD. NT-pro-BNP levels were significantly increased (3,454 pg/mL) in our patient, which relates to the severity of CHD.

In the two-dimensional TTE, the thickening of leaflets, retraction, and reduction of mobility can be observed; three-dimensional (3D) echocardiography helps evaluate valve pathology, allowing a detailed view of structural abnormalities of the valves and subvalvular apparatus [8]. The advantages of 3D over 2D echocardiography include that the former allows the presentation of realistic views of heart valves, enhancing visualization of valvular abnormalities. In addition, 3D multiplanar reconstruction enables the accurate quantification of valvular stenosis or insufficiency severity by measuring the opening and vena contracta areas, respectively [9]. CMRI provides complementary information for diagnosing and monitoring CHD because it evaluates the size of the heart's chambers, the right ventricular function, the degree of valvular regurgitation, and the detection and characterization of cardiac metastasis (incidence of 4%) [10]. CMRI is especially useful when the echocardiogram cannot provide adequate views of the right valves and when valvular abnormalities are not obvious on echocardiography. In addition, quantification of right ventricular volumes and function by CMRI is more accurate than echocardiography. Consequently, this imaging modality is particularly appropriate in asymptomatic or mildly symptomatic patients in the detection of progressive RV dilatation and dysfunction that would encourage early valve intervention [11]. The main clinical application of CMRI in this case was the detection of right ventricular dysfunction, which was not documented in the evaluation by conventional echocardiographic parameters.

Nuclear medicine has grown to play a central role in diagnosing neuroendocrine neoplasia after the identification of somatostatin in 1973 and five types of somatostatin receptors (SSTRs). SSTRs are G-protein-coupled receptors binding to somatostatin neuropeptides, a paracrine secreted by gastrointestinal and brain cells. Various types of somatostatin agonists and few antagonists are available for clinical and/or experimental use. The common radiopharmaceuticals for clinical use are 68Ga-DOTATATE, 68Ga-DOTA-TOC, and 68Ga-DOTA-NOC. These three radiopharmaceuticals differ slightly in their pharmacokinetic properties, mainly due to different affinities for SSTR subtypes. Meanwhile, DOTA-TATE is SSTR 2 specific; DOTA-NOC has an affinity towards SSTR 2, 3, and 5; and DOTA-TOC has an affinity towards SSTR 2 and 5. Well-differentiated neuroendocrine tumors have high expression of SSTR, while poorly differentiated neoplasia show low affinity and high glycolytic activity.

PET/CT has been increasingly used to image NETs. Tracers targeting somatostatin analogs (e.g., ^18^F-AlF- NOTA-octreotide) are the gold standard for lesion detection and even guide the optimal choice of systemic therapies; they highlight well-differentiated cells that express the SSTR. ^18^F-AlF-NOTA-octreotide proved to have the highest in vitro binding affinity for the SSTR. It is a radio-conjugate consisting of octreotide linked to the radionuclide fluorine (F) 18 (^18^F), by the macrocyclic chelating agent, 1,4,7-triazacyclononane-1,4,7-triacetate (NOTA). Fluorine-18 offers several advantages, including a high production yield and a favorable half-life (109.8 min) that allows for centralized production and distribution to distant PET centers without an on-site cyclotron. Moreover, the shorter positron range of ^18^F compared with gallium-68 could improve the spatial resolution of the PET data acquired with modern PET cameras [12]. In addition, positive findings on ^18^F-FDG PET predict aggressive tumor behavior and poorer prognosis. Dual PET/CT imaging provides complementary information about tumor biology and correlates with histological grade. SSTR analog imaging evaluates the presence and extent of somatostatin receptors, whereas ^18^F-FDG uptake reflects glucose metabolism, especially in poorly differentiated NETs. Both tracers act as a prognostic biomarker above histologic grade alone and guide best care [13]. A combined ^18^F-FDG/^68^Ga-DOTATATE PET/CT imaging classification predicts median progression-free survival in patients with metastatic gastroenteropancreatic neuroendocrine neoplasia. This system may have implications for therapeutic management. Patients with all lesions ^18^F-FDG negative and ^68^Ga-DOTATATE positive have a low-grade and indolent disease. In contrast, patients with one or more ^18^F-FDG positive lesions and at least one of them ^68^Ga-DOTATATE negative have high-grade, metabolically active disease and therefore warrant aggressive treatment [14].

The diagnosis of neuroendocrine tumors is characterized by the detection of immunohistochemical markers: synaptophysin, chromogranin A, and neuron-specific enolase. The two most used and currently considered the most specific immunohistochemical markers for neuroendocrine cells and their tumors are chromogranin A (CgA) and synaptophysin (SPY) [15]. CgA is a marker for neuroendocrine secretory granules of four pancreatic hormones and gastrin, while SPY is a marker for synaptic vesicles in neuroendocrine cells, which release classic neurotransmitters, such as acetylcholine and others. CgA is involved in the synthesis and secretion of peptide hormones through exocytosis, while the function of SPY is elusive. CgA immunostaining mostly correlates with each hormone staining in non-β-cell tumors, while SPY immunostaining recognizes endocrine cells diffusely in the cytoplasm.

The Ki-67 index is a measurement of cell proliferation that can be used to predict the long-term recurrence of carcinoid tumors. The Ki-67 is categorized into grades G1 (≤2%), G2 (3-20%), or G3 (>20%) according to the European Neuroendocrine Tumor Society guidelines and the 2010 World Health Organization classification [16] A Ki-67 index of 2% or lower means that fewer than two in every 100 cells (2%) are dividing.

Treatment focuses on controlling and relieving the symptoms of heart failure and CS. Drugs used in heart failure should be used cautiously due to the risk of reducing cardiac output. Somatostatin analogs are the most widely used medical therapy to treat CS because they can control the clinical symptoms of hypersecretion in NETs that express somatostatin receptors, reducing circulating tumor metabolites (including 5-HT). Short-acting analogues (octreotide immediate-release) could be used to stabilize the patient, while longer-acting formulas (octreotide long-acting repeatable (LAR), lanreotide autogel, and lanreotide long-acting) are the standard care in CS [2]. In addition, these agents exhibit antiproliferative activity in vitro. It has been shown that octreotide LAR inhibits tumor growth in patients with metastatic well-differentiated midgut NETs [17]. In the phase III CLARINET trial, the median progression-free survival was 61.5 months, 55.0 months, and 29.7 months for midgut, hindgut, and pancreatic NETs treated with somatostatin analogs, respectively [18]. Valve replacement is reserved for patients with symptomatic severe carcinoid heart disease who have evidence of progressive ventricular dilatation or impaired ventricular function. The choice of prosthesis should be individually tailored according to the bleeding risk, the life expectancy related to the tumor, and potential future interventions. Bioprosthetic valves are preferred due to the increased likelihood of invasive procedures that require temporary discontinuation of anticoagulants, the high risk of bleeding in patients with hepatic dysfunction from carcinoid disease, and the increased risk of thrombosis of right-sided mechanical valves. Patients who have successfully undergone valve replacement with a bioprosthetic valve should receive anti-coagulation therapy with warfarin for three to six months. The perioperative management of patients undergoing valvular surgery for carcinoid heart disease is complex due to the risk of a potentially life-threatening carcinoid crisis. For the prevention of a carcinoid crisis, an octreotide infusion at the dosage of 50 mg/hour is recommended 12 hours before the procedure, throughout the operation, and 48 hours after the surgery, and increased to 100-200 mg/hour, if necessary [10]. A proficient multidisciplinary team integrating cardiologists, oncologists, anesthesiologists, and surgeons with broad experience in the field is required for the complex management of patients with CHD undergoing valvular surgery. Valvular replacement can relieve symptoms of right heart failure and improve outcomes. Reported one- and two-year survival rates after valvular surgery are 56% and 44%, respectively [19].

## Conclusions

This case shows that, even though CHD has a classic presentation, clinical findings can be confusing. Symptoms of CS, such as diarrhea and bronchospasm, are commonly attributed to other gastrointestinal and pulmonary diseases, particularly in young patients. Facial rash has a similar appearance to the butterfly rash seen in systemic lupus erythematosus, leading to misdiagnosis. The case highlights the importance of maintaining a high index of suspicion for rare conditions, such as CHD, in atypical presentations and even more so in patients with signs and symptoms of right heart failure. A multidisciplinary team is needed for the correct diagnosis and treatment. The diagnosis often is the result of the suspicion of internists, while the identification of tumors is often dependent upon radiologists. The detection of heart involvement is typically in the hands of imaging cardiologists, while treatment relies on cardiologists, surgeons, and oncologists. It is crucial to always consider CHD as a differential diagnosis in the presence of primary tricuspid and pulmonary regurgitation. The use of echocardiography and multimodality imaging is vital for prompt diagnosis. Typical findings on echocardiography include thickening, retraction, and reduction of mobility of leaflets, which causes regurgitation and, less frequently, valvular stenosis. 3D echocardiography is particularly useful for enhanced visualization of structural valvular abnormalities and accurate quantification of the severity of valvular lesions. CMRI is crucial to adequately assessing valve pathology, evaluating right ventricular size and function, and detecting metastatic lesions. PET/CT enables the detection of tumoral lesions and provides prognostic information to guide treatment. Early recognition of CS is essential to prevent the development and progression of CHD and its complications, which could improve surgical treatment outcomes. Late mortality after tricuspid and pulmonary surgery is predicted by preoperative functional class, left and right ventricular function, and prosthesis-related complications. Therefore, appropriate timing of intervention is crucial to avoid irreversible right ventricular damage and advanced heart failure and to improve postoperative prognosis.

Areas for future research in CHD include identifying more sensitive and specific biomarkers to predict the presence and severity of CHD, advancements in imaging techniques to better assess valvular abnormalities and their impact on right ventricular size and function, evaluation of prognostic impact of treatment options such as peptide receptor radiotherapy, and developing effective strategies to prevent the occurrence of CHD.
